# An Improved Method for Fabrication of Ag-GO Nanocomposite with Controlled Anti-Cancer and Anti-bacterial Behavior; A Comparative Study

**DOI:** 10.1038/s41598-019-45332-7

**Published:** 2019-06-24

**Authors:** Sadegh Khorrami, Zahra Abdollahi, Ghazaleh Eshaghi, Arezoo Khosravi, Elham Bidram, Ali Zarrabi

**Affiliations:** 1 Department of Biotechnology, Facullty of Advanced Sciences and Technologies, University of Isfahan, Isfahan, Iran; 2 Department of Mechanical Engineering, Khomeinishahr Branch, Islamic Azad University, Khomeinishahr/Isfahan, Iran; 3 Department of Chemical and Biomolecular Engineering, University of Melbourne, Melbourne, VIC 3010 Australia; 4 Sabanci University, Nanotechnology Research and Application Center (SUNUM), Tuzla, Istanbul, 34956 Turkey

**Keywords:** Breast cancer, Nanostructures, Biomedical materials

## Abstract

In this study, two green procedures for Silver-Graphene Oxide (Ag-GO) nanocomposite synthesis were investigated. As a common method, AgNO_3_ was first loaded on the GO surface and then was reduced and stabilized by walnut green husk extract, producing Ag-GO-І. As an innovative approach, GO was first exposed to the extract and then the AgNO_3_ was added as the second step, producing Ag-GO-П. Physicochemical properties, antibacterial and cytotoxicity activity of both nanocomposites were subsequently studied comparing with free silver nanoparticles (AgNPs) and pure GO. Based on the results, exposure of GO to the extract, as a reducing agent, at the first/last step of the synthesis process resulted in the fundamental differences in the final products. So that, high amounts of agglomerated silver nanoparticles were formed between the GO sheets, when using the common method, whereas in Ag-GO-П, small AgNPs were formed on the GO sheets without aggregation, entirely covering the sheets. Antibacterial and cytotoxic behavior of these nanomaterials could be compared as AgNPs > Ag-GO-П > Ag-GO-І. It is assumed that these differences are due to control of unwanted nucleation in the synthesis process that Ag nanoparticles are smaller with less agglomeration when the GO surfaces are pre-treated with reducing agent.

## Introduction

During the last decade, nanotechnology has witnessed eye-catching growth spreading in almost all aspects of scientific and industrial research. Nonetheless, this development has never been free of challenges and failures^1–4^. The most important and outstanding challenges and limitations associated with this new and promising technology is the environmental concerns associated with the production and application of nanostructures^5–8^. The main causes of this concern are the chemical and physical procedures requiring high degrees of energy-consumption and costs, as well as carrying greatly-toxic chemicals endangering human beings and the environment^9–12^. Hence, the researchers are greatly motivated to replace conventional procedures with more appropriate ones.

A wide range of attempts has been made to present novel procedures based on green chemistry principles^13^. In this approach, natural and non-toxic agents, such as the plant extracts, bacteria, fungi, and unicellular microorganisms are applied as the reducing and stabilizing agents in the process of nanoparticle’s production^14^. In addition to the straightforward, cheap and environment-friendly procedure, it is able to reduce the biomedicine application-related concerns^10,13,15,16^. Despite myriads of advantages, green synthesis of nanoparticles faces a number of limitations, including the low yield and lack of sufficient control over the process of synthesis^17^. As an example, although the plant extracts function properly as a reducing and stabilizing factor, controlling the shape and arrangement of nanoparticles is unlikely^17,18^.

Silver is one of the noble metals utilized by human beings considering its prominent biological, catalytic, optical and electromagnetic properties^19^. Silver nanoparticles (AgNPs) enjoy unparalleled biological properties, such as the antimicrobial, antifungal, anti-inflammatory, antioxidant and anticancer properties^20–23^. Notably, the desired properties of this functional metal element are improved in nanometric dimensions^24^. However, the unruly activities of the silver nanoparticles in biological systems result in the undesirable side effects as the major limitation of their applications^25–27^.

Nowadays, metal nanoparticle composites, especially graphene oxide (GO)-silver (Ag) nanocomposites, are considered as the most admired nanostructures, as this makes the biological activity of silver nanoparticles under control^28,29^. The high specific surface area of the GO sheets makes them an appropriate platform for entrapping silver nanoparticles^24,30,31^. Once the silver nanoparticles are entrapped between the GO sheets, the possibility of their agglomeration significantly declines while their release and free movements are extremely restricted^32–34^. It is then expected that the combination of silver nanoparticles with GO sheets have more desirable biological functions compared to any of the nanomaterial alone.

Earlier, Alsharaeh *et al*. used lemon juice under microwave irradiation (MWI) and UV light irradiation for synthesizing AgNPs/reduced graphene oxide (rGO) nanocomposites. The Ag NPs with a size range from 3 nm to 8 nm dispersed on the rGO sheets^32^. Korsi *et al*. prepared AgNPs/rGO with using a wet chemical method. Ag nanoparticles with spherical shape and an average size of about 6–10 nm decorated on the rGO sheets^35^. Gurunathan *et al*. synthesized silver nanoparticle/graphene oxide nanocomposite in the presence of AgNO_3_ and pepsin. The AgNPs with an average size of 20 nm distributed on the GO sheets^36^. Bozkurt synthesized Ag-graphene nanocomposites by the one-step sonochemical method and used sodium citrate as a green reducing agent. The average size of the spherical Ag nanoparticles was approximately 20 nm^37^. Linh *et al*. used glucose, as a green reducing and a crosslinking agent, by a one-pot hydrothermal process to produce Ag nanoparticle with size about 50–100 nm that decorated rGO^38^. Anyway, compared with these previous reports, in the present study walnut green husk extract just used as reducing and stabilizing agent. Moreover, our recent investigation revealed this extract has anticancer activity^23^. It is worth mentioning that walnut green husk is normally known as agricultural waste, therefore in contrast with other agents previously used, the walnut extract not only is completely cheap and available but also is a pharmaceutical agent naturally.

In addition to what was mentioned above, in this study, two green procedures of Ag-GO nanocomposites synthesis were investigated and the results are compared. In a common method, the silver precursor, silver nitrate, was first loaded onto the surface of GO, the silver nitrate was then reduced and stabilized by the reducing agent (Ag-GO-І)^33,39–42^. As an innovative approach, GO was first exposed to the reducing agent and then the silver nitrate was added as the second step producing (Ag-GO-П). Physicochemical properties, antibacterial and cytotoxicity activity of both nanocomposites were subsequently studied.

## Materials and Methods

All reagents used in this research were analytical grade and were used without further purification or treatment. High-purity chemical reagents (graphite powder, silver nitrate (99.98%), sulfuric acid (H_2_SO_4_) 95%, phosphoric acid (H_3_PO_4_) 95%, nitric acid (HNO_3_) 65%, hydrogen peroxide 30%, hydrochloric acid (HCl) 37%, ethanol (≥99.5%), anhydrous dimethyl sulfoxide (DMSO), and potassium permanganate (KMnO_4_) were purchased from Merck and Sigma-Aldrich chemical companies. Standard bacterial strains of *Escherichia coli* (35218 ATCC) *Pseudomonas aeruginosa* (1214 PTCC) and *Staphylococcus aureus*, (1189 ATCC), and breast cancer cell line (MCF-7) were purchased from Pasteur Institute, Iran.

### Preparation of GO

The modified Hummer’s method introduced by Islami *et al*. was used to produce GO^30^. In brief, 0.5 g of graphite powder was formerly treated with the specified volume ratio (3:1) of the concentrated acids (H_2_SO_4_:HNO_3_) for 24 hours at room temperature. 120 mL of DH_2_O (deionized water) was then added to the solution quenching the reaction. The consequential product was then washed out with DH_2_O and freeze-dried subsequently. Afterward, 0.5 g of the obtained product was mixed with H_2_SO_4_ (110 mL), while 3 weight equivalents of KMnO_4_ (1.3 g) was slowly added during 1 hour. To maintain the reaction temperature below 20 °C, the ice water bath was used. The subsequent solution was stirred for another 24 hours at room temperature. 300 mL DH_2_O was then added to the solution resulting in an exothermic reaction, while the temperature of the mixture dropped to 25 °C after half an hour. Adding an aqueous solution of H_2_O_2_ (a 3:1 volume ratio) makes the reaction terminated changing the solution’s color to yellow–brown. This product was then centrifuged at 4,000 rpm for 10 minutes, washing out with HCl (37%) and DH_2_O.

### Synthesis of Ag-GO Nanocomposits

Ag-GO-І was synthesized according to the commonly reported methods. Briefly, 25 mg of GO was dissolved in 50 mL DH_2_O, and ultra-sonicated for 45 minutes using a water bath sonicator. It was then mixed with 50 mL AgNO_3_ (final concentration = 6 mM) and the sonication was continued for 15 more minutes. 50 mL of walnut extract as a reducing and stabilizing agent was then gradually added to the mixture, while the pH was adjusted at 8 using NaOH, and it was vigorously stirred.

In order to synthesize Ag-GO-П, a similar amount of GO solution was ultra-sonicated for 45 min. Afterward, it was mixed with 50 mL of the extract (pH = 8) and sonicated for another 15 min. 50 mL of the AgNO_3_ solution (final concentration = 6 mM) was then gradually added to the mixture under vigorous stirring.

Both mixtures were stirred for 24 h at 30–35 °C in the subsequent similar condition.

### Characterization of Ag-GO nanocompunds

#### UV-Visible Spectroscopy

The UV-Visible spectroscopy (Mettler Toledo V 670, United States) was used to assess the effect of time on the formation of Ag nanoparticle on the GO surface within the range of 300–700 nm. Optical properties of the sensitive materials to concentration, size, shape and agglomeration state, make the UV-Visible spectroscopy valued to identify metal nanoparticles. Detecting the specific peak at the precise wavelengths would be due to the surface plasmon resonance of the electrons present on the nanoparticle surface.

#### XRD

Crystallographic studies of both synthesized nanocomposites were analyzed using an X-ray diffractometer (XRD, D8 Advance, Germany) with Cu Kα (λ = 1.5405 Å) as a radiation source within 2θ = 10–80° at the scan speed 0.4°/min. The acquired peaks from the XRD analysis were used to recognize the chemical structure and crystalline configuration of the material.

#### FT-IR

To validate the potential effect of walnut green husk extract on the surface modification of the GO and synthesized nanocomposites, Fourier Transform Infrared spectroscopy (FT-IR) was performed using JASCO 6300, Tokyo, Japan.

#### FE-SEM, EDS

To investigate the morphology and any probable agglomeration of the Ag nanoparticles, as well as assembling of these nanomaterials, the field emission scanning electron microscopy (FESEM) (MIRA3 TESCAN, Czech Republic) was used. The chemical composition of nanoparticles was assessed using energy-dispersive X-ray spectroscopy (EDS) (using the same instrument).

### Biological behavior

#### Antibacterial study

The antibacterial activity of the synthesized nanocomposites was investigated by agar well diffusion method against the standard strains of *Escherichia coli*, *Pseudomonas aeruginosa*, and *Staphylococcus aureus*. In a typical test, the bacterial strains were grown on Mueller Hinton Broth (MHB) medium at 37 °C for 18 h. Then, their 0.5 McFarland standard suspension was prepared. The strains were then swabbed onto Mueller Hinton Agar (MHA) plates, and wells were formed by punching the medium using a sterilized Pasteur pipette. 60 μL of the equivalent concentration of each compound was then added to each well for a rational comparison, and the plates were incubated at 37 °C for 18 h. The diameter of the inhibition zone was measured in mm using an accurate digital ruler. MIC and MBC were also investigated using the standard broth dilution method. Briefly, 50 μL of overnight grown bacterial cultures (0.01 of 0.5 McFarland) along with 100 µL uncultured MHB were placed into 96 well plates. Then, 100 μL of each composite by serial dilutions of 5–100 μg/mL final concentrations were added to the plates and incubated at 37 °C for 18 h. Wells containing culture media and the bacterial suspension was considered as control. To confirm the bacterial death, the wells showing no visible growth were swabbed on MHA plates and were incubated for 18 h at 37 °C.

#### Cytotoxicty study

To determine the cytotoxicity of both synthesized nanocomposites, GO and modified GO, free AgNPs and walnut extract, the cell viability was studied using the conventional MTT colorimetric assay. Briefly, MCF-7 cells were seeded in 96 well plates at the density of 5000 cells/well in the presence of 100 μL cell culture medium (DMEM supplemented with 10% FBS and 1% penicillin–streptomycin solution). Cells were incubated for 24 h in an incubator containing 5% CO_2_ at 37 °C. After 24 h, the existing media was replaced with the fresh media along with the various concentrations of the compounds, incubated for 24–72 h at 37 °C. To study the cell viability, the cell containing media was replaced with 100 μL pure medium. 10 µL of MTT solution (5 mg/mL in DMSO) was afterward added to each well, and the plates were incubated for another 4 h. The MTT solution was then discarded, while 100 μL of DMSO was added to each well followed by incubation for another 40 min in dark conditions. The solution was pipetted and its absorbance was then recorded at 492 nm using a microplate reader (Bio-Rad, USA).

### Statistical analyses

The data were exposed to One-way Analysis of Variance (ANOVA) to determine the importance of individual differences at p < 0.05 level. Important means were compared by Duncan’s multiple range tests. All statistical analyses were performed using SPSS Version 16.

## Results and Discussion

### Characterization

#### UV-Visible spectroscopy

**Figure 1:**
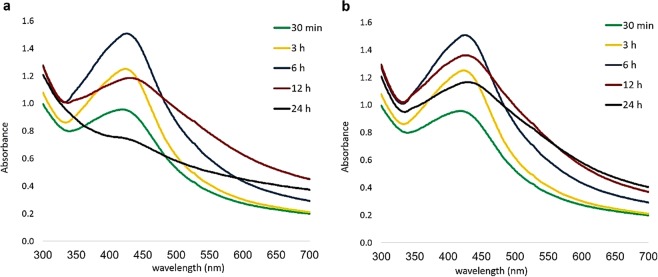
UV-Visible spectra of the colloidal Ag-GO nanocomposites (**a**) Ag-GO-І and (**b**) Ag-GO-П indicating the increase, and subsequently decrease in the peak intensity along with a redshift with the passing of time.

UV−Visible spectra of GO-Ag nanocomposites (Fig. 1) show the successful formation of AgNPs on GO surfaces. Considering that the surface plasmon resonance (SPR) peak at around 425 nm in both mixtures^33,43,44^, the absorption bands gradually shifted to the longer wavelength (Red Shift) during 24 h. The surface plasmon band shifting depends on the particle size, shape, chemical surrounding, adsorbed species on the surface and dielectric constant^43,45–47^. This may illustrate the formation of larger AgNPs, with different shapes and sizes.

Clearly, the intensity of the absorption peaks went up over time, while this process is reversed after 6 hours, when the absorption intensity went down. Based on the spectra, the decreasing absorption of Ag-GO-І is more than the Ag-GO-П. It may occur due to the agglomeration of AgNPs in Ag-GO-І^42^. It seems that the exposure of GO to the walnut extract, as a reducing agent, prohibits the agglomeration of the obtained AgNPs on the GO surface.

#### X-ray diffraction analysis

**Figure 2:**
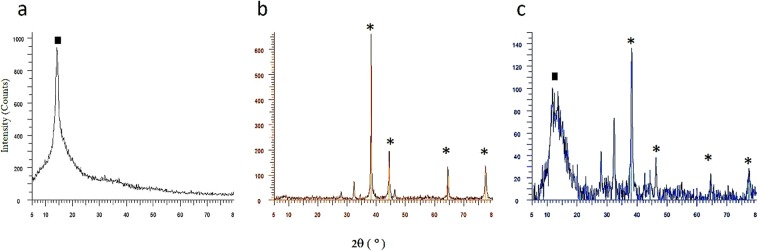
XRD spectrum of (**a**) pure GO, (**b**) Ag-GO-І and (**c**) Ag-GO-П. Peaks assigned with an asterisk (*) represent the Silver, and the black square (■) corresponds to the Graphene Oxide.

Appearing a characteristic peak at 2θ = 15° in the XRD spectrum belong to GO sheets confirms this product have been synthesized successfully (Fig. 2a). The presence of AgNPs in both nanocomposites was also confirmed by the XRD analysis. The detected peaks at 2θ = 38.2°, 44.3°, 64.6° and 74.6° are attributed to the (111), (200), (220) and (311) crystalline planes of cubic silver nanoparticles (code; 01-087-0720), respectively. However, the characteristic peak of GO sheets disappeared after the sheets were decorated with silver nanoparticles in Ag-GO-I. This means that, the anchoring of silver nanoparticles on the surface of the GO sheets prevents the stacking of the GO layers^48,49^. It is when, the XRD spectrum of the Ag-GO-II (Fig. 2c), in addition to the characteristic peaks of silver nanoparticles, shows the GO related peaks appeared with lower intensity and more expansion than the pure GO. According to the Scherrer Equation (Eq. 1), the 4.2 nm space between GO sheets decreased to 1.67 nm^50^. This phenomenon refers to the restacking of GO sheets due to the reduction of the existing functional groups on GO, subsequently covering by AgNPs^40,43,51^.

1 $$D=\frac{0.9\lambda }{\beta cos\theta }(nm)$$

With *D* being the crystallite size, λ the X-ray wavelength, *β* the full peak width at half-maximum (FWHM), and *θ* representing the Bragg angle.

#### FT-IR analysis

**Figure 3:**
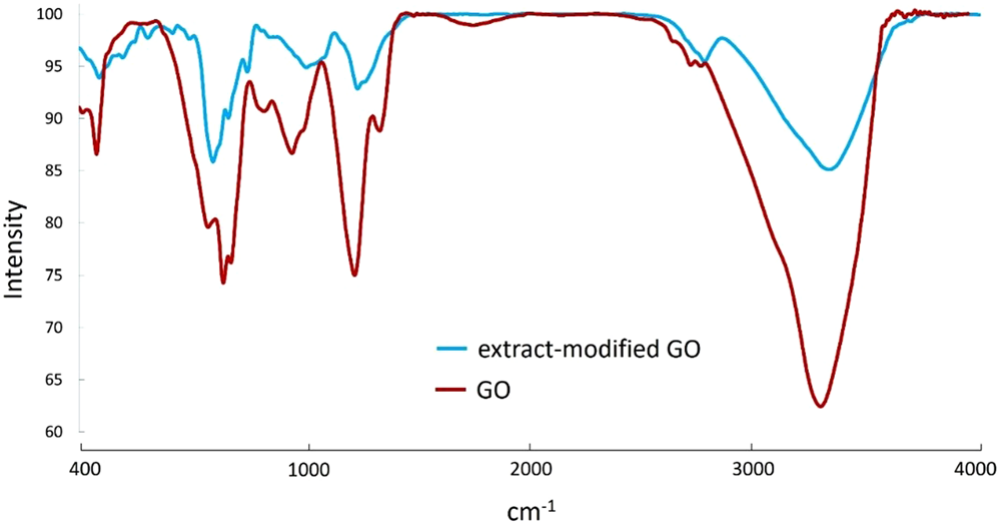
FT-IR spectra of the pure GO and GO exposed to Walnut green husk extract. The lower FT-IR peak intensity of the extract modified GO than pure GO confirms the reduction of functional groups in this compound.

As shown in Fig. 3, the absorption peak at 3427 cm^−1^ is attributed to OH stretching groups, evidently detected at both GO and the extract modified GO (MGO). The identified bands at 1714 cm^−1^, 1620 cm^−1^, 1377 cm^−1^ and 1063 cm^−1^ corresponded to the C=O, C=C, epoxy C-O groups and alkoxy C-O, respectively. However, A distinct decrease is appeared in the intensity of modified GO peaks compared to the pure GO confirming the reduction of GO functional groups after exposing to the extract^24,37,52,53^.

#### FE-SEM, EDX

**Figure 4:**
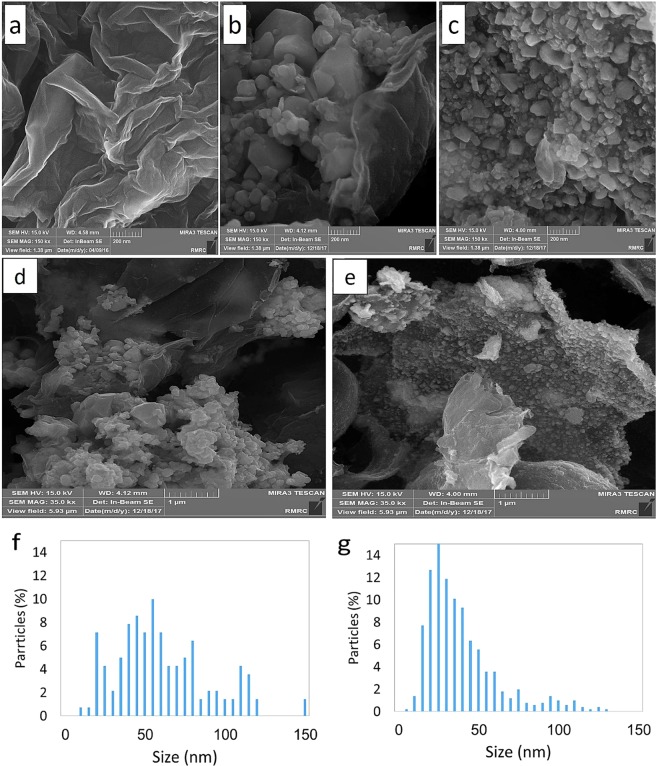
Representative field emission scanning electron microscopy (FESEM) images of pure GO (**a**), Ag-GO-I (**b**,**d**) and Ag-GO-II (**c**,**e**) as well as their particles size distribution, (**f**,**g**), respectively.

Field Emission Scanning Electron Microscopy (FE-SEM) was used to analyze the morphological aspects, size distribution and chemical composition of pure GO and both Ag-GO nanocomposites. The results of characterizations conducted by Islami *et al*. revealed that the thickness and diameter of individual GO sheets synthesized using the present method were <1 nm and ~0.3–0.7 μm, respectively, and most of the synthesized GO sheets were single-layered^30^. Fig. 4a shows the FE-SEM image of pure GO sheets. The FE-SEM images of the composites, not only confirmed the results of the XRD and UV-Visible spectroscopy, but it also shows that the silver nanoparticles are formed anisotopically. Different sizes and shapes of AgNPs, such as cubic, triangular, multifaceted, and spherical form on the surface of GO are obviously seen in Fig. 4b,c. As Fig. 4b shows, the agglomerated silver nanoparticles are randomly embedded into the GO sheets in Ag-GO-І with the average size of 67 ± 34 nm, while in Ag-GO-П, the relative small silver nanoparticles (41 ± 20 nm) cover the whole surface of GO sheets (Fig. 4d,e). In addition, the size distribution of the nanoparticles in Ag-GO-І is highly border than Ag-GO-П (Fig. 4f,g).

Based on our previous protocol for the spherical silver nanoparticles synthesis utilizing the extract^23^, presence of GO nanosheets seems to act as a morphological driver for AgNPs, dictating the formation of polymorph nanoparticles in the Ag-GO nanocomposite^22,44^. The size and shape of the Ag nanoparticles are also influenced by the concentration of AgNO_3_ solution^45^.

**Figure 5:**
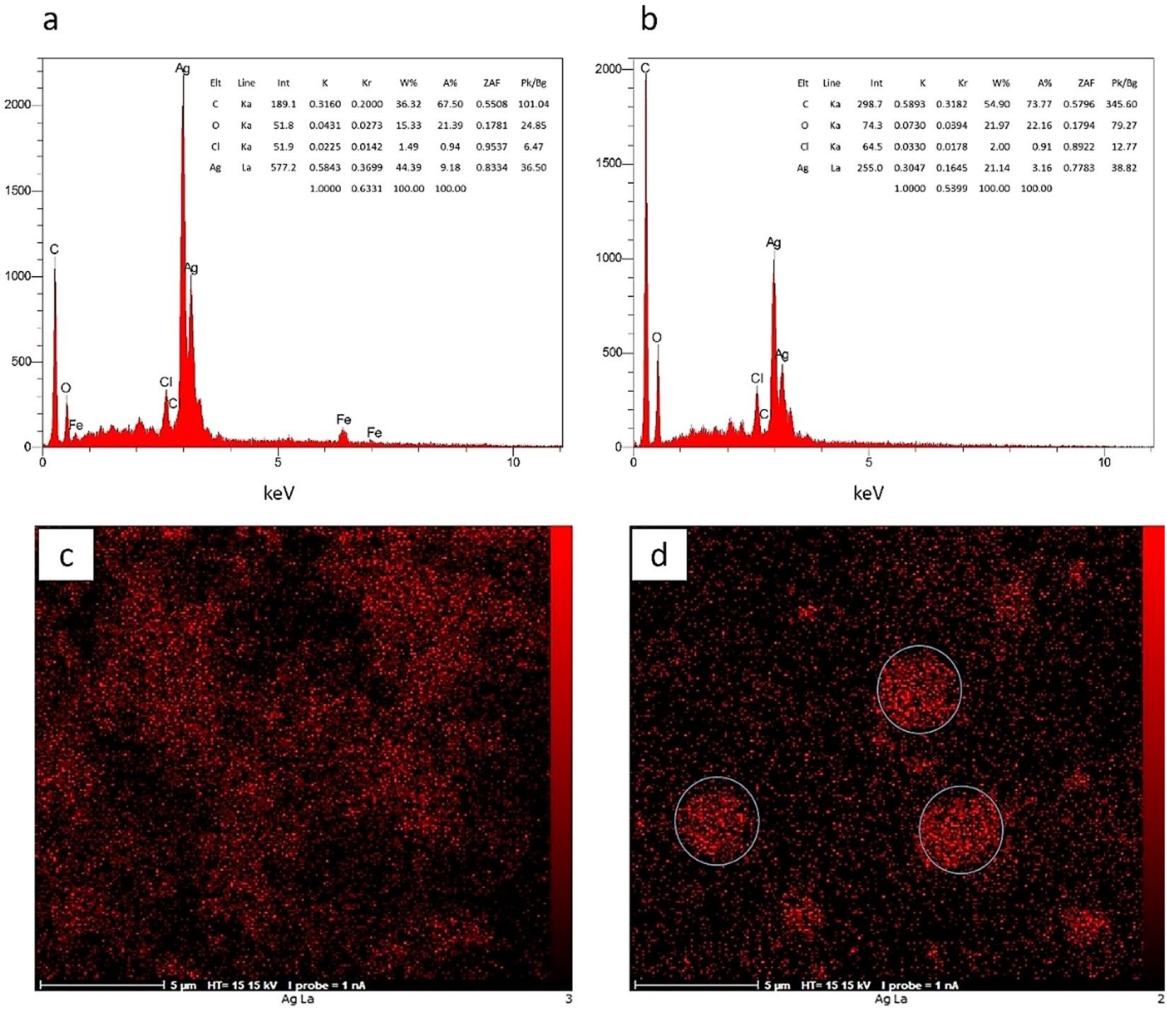
EDS spectrum of (**a**) Ag-GO-І and (**b**) Ag-GO-II, showing differences of the amount of silver and carbon elements in two synthesized nanocomposites, and EDS-map of (**c**) Ag-GO-І and (**d**) Ag-GO-II.

The presence of AgNPs on GO sheets was also elementally identified with EDS analysis (Fig. 5). As the data show, the weight percent of silver (44.39%) is higher than carbon (36.32%) and oxygen (15.33%) as the main elements of the synthesized Ag-GO-І. This could be another evidence for agglomeration of nanoparticles in the nanocomposite. In the Ag-GO-П, however, the weight percent of carbon (54.90%) and oxygen (21.97%) is more than silver (21.14%), the equivalent (similar) concentrations of silver nitrate and GO has been used in the synthesis process of both compounds.

The main reason for this difference might be due to the limitation of the GO functional groups after the treatment with walnut green husk extract^40^. In other words, deactivation of the functional groups, which could enhance inappropriate nucleation of AgNPs, prevents the redundant formation of the nanoparticles on GO surfaces^42,54^. Therefore, when the surface of the functionalized GO sheets saturated with a single layer of AgNPs in the Ag-GO-П, more NPs are not able to be formed on the GO surface. Hence, the relative amount of silver in this composite is less than the Ag-GO-І. Notably, metal nanoparticles, such as Ag and Au, can interact with the GO sheets through physisorption, electrostatic binding, or through charge-transfer interactions^39,55^.

Furthermore, EDS-map results demonstrate a better dispersion of nanoparticles in Ag-GO-П (Fig. 5d) than Ag-GO-І (Fig. 5c). Indeed, some local agglomeration of AgNPs observed in the Fig. 5d confirm this assumption that in the Ag-GO-П, after saturation of GO sheets, no excess AgNPs are allowed to connect to the GO sheets and the surplus nanoparticles inevitably aggregate to each other.

### Biological studies

Due to the observed physicochemical differences in the derived nanocomposites from two approaches, various biological properties is expected. To investigate this assumption, the antibacterial and cytotoxic properties of both compounds were evaluated and compared with the free silver nanoparticles synthesized using the extract, as well as the naked GO sheets.

#### Antibacterial activity

**Figure 6:**
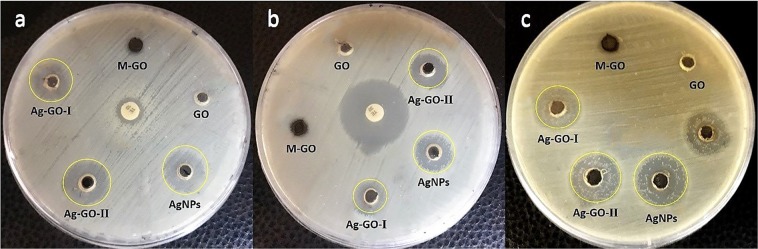
Antibacterial activity of pure GO, M-GO, Ag-GO-І, Ag-GO-П and free AgNPs against (**a**) *E. coli*, (**b**) *P. aeruginosa*, (**c**) *S. aureus*.

Considering the results of antibacterial studies (Fig. 6 and Table 1), the investigated silver-based nanomaterials have an applicable antibacterial activity against both gram positive and gram negative types of bacteria. However, the antibacterial activity of free AgNPs is evidently more than that of Ag-GO-П, and much greater than that of Ag-GO-І (AgNPs > Ag-GO-П > Ag-GO-І). Similar to the previous reports, a non-toxic behavior was observed from the pure GO and modified GO (MGO)^56,57^. Hence, the antibacterial activity of the synthesized nanocomposites could be solely attributed to the presence of silver nanoparticles which represents that high level of antibacterial activity of Ag-GO-П is rational. Since theses nanoparticles are placed on the surface of GO sheets, they are directly exposed to the bacterial cells. As illustrated in Fig. 4, the GO platform prevents the agglomeration and enlargement of silver nanoparticles, improving the performance of the composite.

**Table 1:** Diameter of inhibition zone (DIZ) (mm) and minimum inhibitory concentration (MIC) (µg/mL) of modified GO (MGO), pure GO, Ag-GO-І, Ag-GO-П and AgNPs against different bacterial species.

	M-GO/GO		Ag-GO-І		Ag-GO-П		AgNPs	
	DIZ	MIC	DIZ	MIC	DIZ	MIC	DIZ	MIC
*S. aureus* 1189 ATCC	—	—	13	40	18	25	15	20
*P. aeruginosa* 1214 PTCC	—	—	15	30	18	15	18	10
*E. coli* 35218 ATCC	—	—	18	30	20	10	22	5

The stabilization of silver nanoparticles on the surface of the GO, however, controls the release of these nanoparticles in the environment^34,43,58^. This is considered as the main reason for the lower antibacterial activity of this nanocomposite compared with the free silver nanoparticles.

In the Ag-GO-І, the nanoparticles are surrounded by GO sheets limiting their freedom, less exposed than nanoparticles of the Ag-GO-П, thus presenting a lower antibacterial activity. The aggregation of AgNPs also reduces the activity of this compound^21,59,60^.

#### Cytotoxicity study

**Figure 7:**
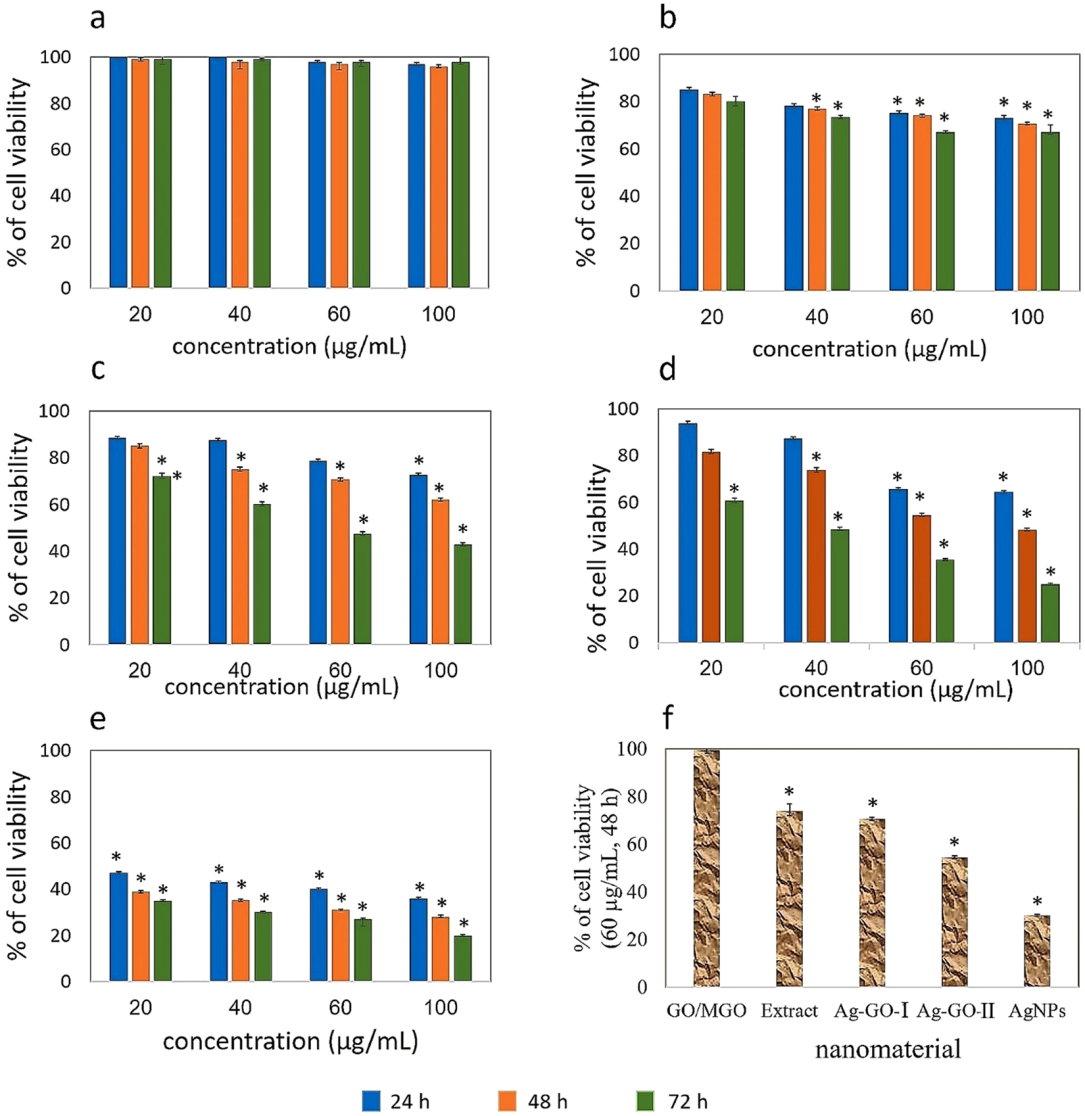
Viability of MCF-7 cells after 72 hours exposure to 20–100 µg.ml^−1^ of (**a**) GO or MGO, (**b**) walnut green husk extract, (**c**) Ag-GO-І, (**d**) Ag-GO-П and (**e**) AgNPs. (**f**) shows a summary comparison of cytotoxic activity of the nanomaterials after 48 hours exposure to 60 µg.ml^−1^. Triplicate incubations for each treatment were conducted in each independent experiment. P values were calculated using one-way ANOVA test (*P < 0.05).

To assess the cytotoxicity of the synthesized nanocomposites, the toxic activity of the compounds against MCF-7 tumor cells was evaluated over the 72 hours using MTT colorimetric assy. As Fig. 7 shows, despite the significant reduction in the cells’ survival affected by both compounds, this reduction is greater for Ag-GO-П than that of the Ag-GO-І. This composite reduces the cell viability by 36, 52 and 75% at a concentration of 100 μg/mL after 24, 48 and 72 hours, respectively, while the Ag-GO-І reduces the cells’ viability by 28%, 38% and 57% during the same time. As Fig. 7e shows, the free silver nanoparticles demonstrate higher levels of cytotoxicity, since they reduced the viability of cells by 80% in 72 hours. It is worth mentioning that GO did show no toxicity against the treated cells during the same periods of time (Fig. 7a).

## Conclusion

The results of this study revealed that the order of the steps in the Ag-GO nanocomposites fabrication could be influential in the size and spatial formation of Ag nanoparticle on the GO surface. We deem that it is due to control of unwanted nucleation and growth in the synthesis process that Ag nanoparticles are smaller with less agglomeration when the GO surfaces and pre-treated with reducing agent. In addition, the results demonstrated that how different assembly of AgNPs within GO nanosheets could greatly affect the biological properties of the nanocomposites. Thus, this method could be introduced as an applicable procedure for the synthesis of different metal nanoparticles on the graphene oxide sheets possibly providing the various forthcoming medicinal, industrial and technological applications.
